# Transcatheter aortic valve replacement before to breast cancer management: case report and literature review

**DOI:** 10.1093/ehjcr/ytae475

**Published:** 2024-09-03

**Authors:** Heberto Aquino-Bruno, Roberto Muratalla-González, Juan F Garcia-Garcia, Julieta D Morales-Portano, Gabriela Meléndez-Ramírez, Yusihey Ahu-Chandomi, Jose A Merino-Rajme, Marco A Alcantara-Meléndez

**Affiliations:** Interventional Cardiology Service, Centro Médico Nacional 20 de Noviembre, Av. Felix Cuevas #540, Col. Del Valle Del. Benito Juarez, Mexico City 03100, Mexico; Interventional Cardiology Service, Centro Médico Nacional 20 de Noviembre, Av. Felix Cuevas #540, Col. Del Valle Del. Benito Juarez, Mexico City 03100, Mexico; Interventional Cardiology Service, Centro Médico Nacional 20 de Noviembre, Av. Felix Cuevas #540, Col. Del Valle Del. Benito Juarez, Mexico City 03100, Mexico; Echocardiography Service, Centro Médico Nacional 20 de Noviembre, Av. Felix Cuevas #540, Col. Del Valle Del. Benito Juarez, Mexico City 03100, Mexico; Cardiovascular Imaging Service, Centro Médico Nacional 20 de Noviembre, Av. Felix Cuevas #540, Col. Del Valle Del. Benito Juarez, Mexico City 03100, Mexico; Pathology Service, Hospital General Zona 1 ‘Nueva Frontera’ IMSS, Carretera costera Huixtla-Tapachula y calle Poniente S/N, CP 30767, Tapachula Chiapas, Mexico; Interventional Cardiology Service, Centro Médico Nacional 20 de Noviembre, Av. Felix Cuevas #540, Col. Del Valle Del. Benito Juarez, Mexico City 03100, Mexico; Interventional Cardiology Service, Centro Médico Nacional 20 de Noviembre, Av. Felix Cuevas #540, Col. Del Valle Del. Benito Juarez, Mexico City 03100, Mexico

**Keywords:** Case report, Aortic stenosis, TAVR, Breast cancer, Chemotherapy

## Abstract

**Background:**

The coexistence of aortic stenosis (AS) and neoplastic pathology are common due to shared risk factors with atherosclerotic disease, such as diabetes, inflammatory conditions, and smoking. Severe AS in patients with cancer requires careful assessment in order to select the appropriate therapeutic choices and their timing (i.e. valve treatment first vs. cancer treatment first).

**Case summary:**

A 66-year-old woman with a history of smoking was admitted to our centre due to heart failure (HF). During her hospitalization, severe AS with severe ventricular dysfunction and cancer were documented. Because of her severe heart disease, she was unable to receive antineoplastic treatment. Therefore, she underwent percutaneous surgery to treat the aortic valve. After that, the management of cancer became possible, which included bilateral radical mastectomy and chemotherapy.

We are presenting a case of cancer coexisting with aortic stenosis and reduced left ventricle ejection fraction. In this case, we performed Transcatheter Aortic Valve Replacement (TAVR) with the aim of improving the ejection fraction, followed by chemotherapy.

**Discussion:**

Cancer patients may be further disadvantaged by AS if it interferes with their treatment by increasing the risk associated with oncologic surgery and compounding the risks associated with cardiotoxicity and HF. Clinical trials and guidelines on TAVR exclude cohorts with limited life expectancy. Hence, the correct and optimal care for cancer patients with severe AS is complex. The TAVR, for cancer patients with severe AS, can more frequently be the best clinical choice by avoiding cardiopulmonary bypass, minimal invasiveness, and therefore, shorter recovery time.

**Learning points**

- The coexistence between aortic stenosis and neoplastic diseases makes decision-making a challenge about which disease should be treated first.
- The concomitant presence between aortic stenosis and neoplastic disease is high because they share the same risk factors. Therefore, when a patient undergoes transcatheter aortic valve replacement (TAVR), screening for neoplasia should be performed intentionally.
- Patients with aortic stenosis and a localized cancer have a high-risk of cardiotoxicity, so TAVR can be considered after the exclusion of metastatic disease, with the aim of improving left ventricle ejection fraction prior to chemotherapy management.

## Introduction

Aortic stenosis (AS) is occasionally diagnosed in cancer patients undergoing cardiovascular evaluation. Likewise, cancer is often recognized during assessment preceding aortic valve interventions.^1^ The coexistence between AS and neoplastic pathology is frequent because they share the same risk factors as atherosclerotic disease (hypertension, obesity, diabetes, smoking, and dyslipidaemia).^2,3^

Cancer patients may be further harmed by AS due to interference with their treatment by increasing the risk associated with oncologic surgery and aggravating the risks associated with cardiotoxicity and heart failure (HF).^4^ Clinical trials and guidelines on transcatheter aortic valve replacement (TAVR) exclude patients with limited life expectancy. On that account, the correct and optimal care for cancer patients with severe AS is complex.^5^

Severe AS in patients with cancer requires careful assessment in order to select the appropriate therapeutic choices and their timing (i.e. valve treatment first vs. cancer treatment first).^1,4^ Surgical valve replacement, transcatheter valve implantation, balloon valvuloplasty, and medical therapy are possible treatments for aortic valve stenosis. However, when malignancy is present, the choice between these options must take into account the stage of cancer and associated treatment, expected outcome, and comorbidities.^1^

We present a case of breast cancer coexisting with severe AS and reduced left ventricle ejection fraction (LVEF), where we performed TAVR with the aim of improving the ejection fraction to subsequently perform the cancer treatment.

## Summary figure

**Figure:**
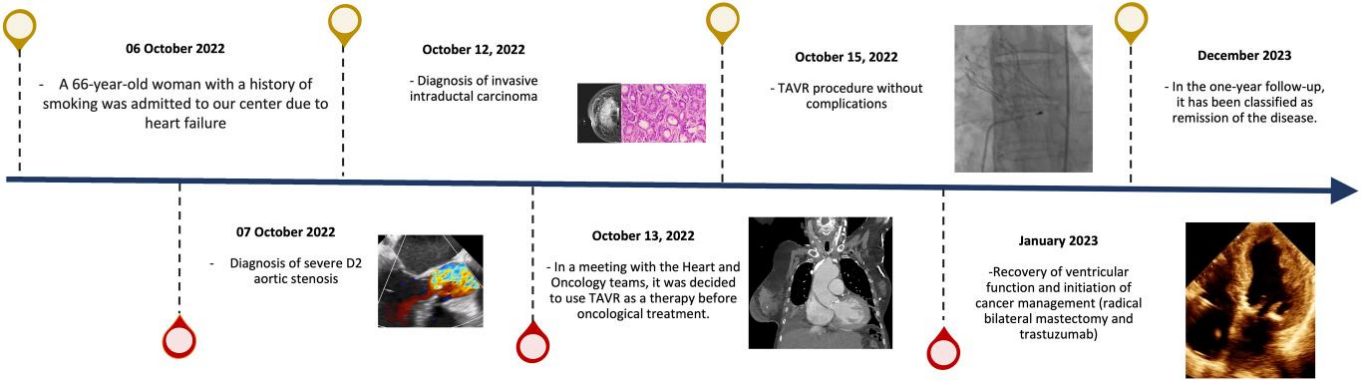


## Case description

**Figure 1:**
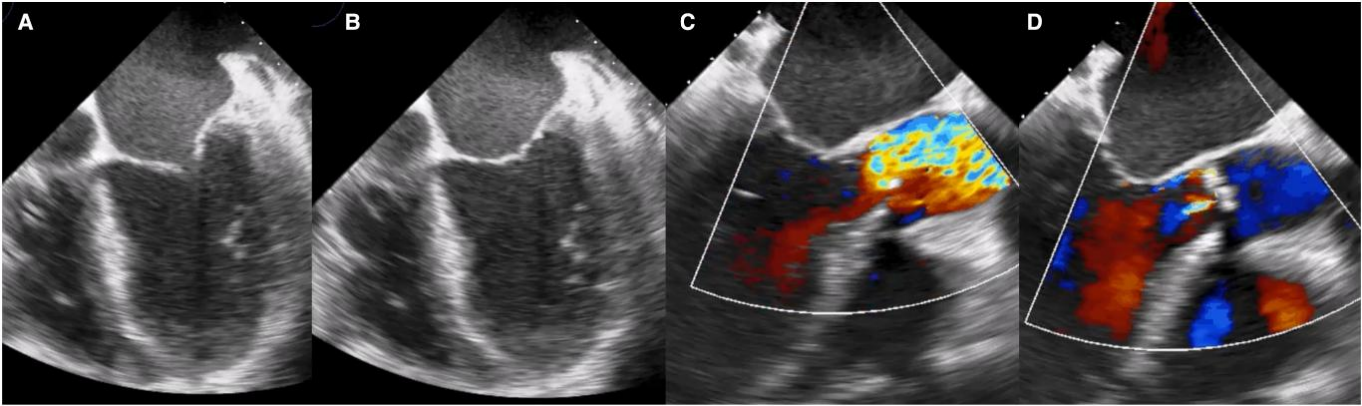
Transoesophageal echocardiogram showing the left ventricle with decreased generalized mobility, (*A*) ventricular systole, (*B*) ventricular diastole, (*C*) turbulent flow secondary to valve obstruction is observed, and (*D*) mild aortic insufficiency.

A 66-year-old woman, with a history of smoking for 35 years, reported an increase in the volume of her right breast 1 year prior to admission. However, she had not undergone any prior medical evaluation. She was admitted to our hospital due to acute HF. She reported orthopnoea and paroxysmal nocturnal dyspnoea of 3 months’ duration. Upon admission, vital signs were as follows: blood pressure of 107/67 mmHg, heart rate 105 b.p.m. On physical examination, a systolic murmur in the aortic focus was heard. The results of the biomarkers were NT-proBNP 5148 pg/mL (< 125 pg/mL) and troponin 1.4 ng/mL (< 1.9 ng/mL). For the diagnostic approach, a transthoracic echocardiogram was performed, which demonstrated severe AS (mean gradient 37 mmHg, velocity 3.8 m/s, area 0.8 cm, index area 0.4 cm/m^2^, LVEF 22%, pulmonary artery systolic pressure (PSAP) 60 mmHg) (*Figure 1A–D* and Supplementary material online, Video S1 of supplementary material). The flow reserve reported an increase of more than 20% in stroke volume in response to low-dose dobutamine. We initiated medical treatment for acute HF with low-dose loop diuretic therapy (furosemide 10 mg every 6 h i.v.), to avoid vasodilation. When the clinical condition improved, we discontinued the loop diuretic and started metoprolol 25 mg every 12 h, enalapril 2.5 mg every 12 h, and spironolactone 25 mg every 24 h.

**Figure 2:**
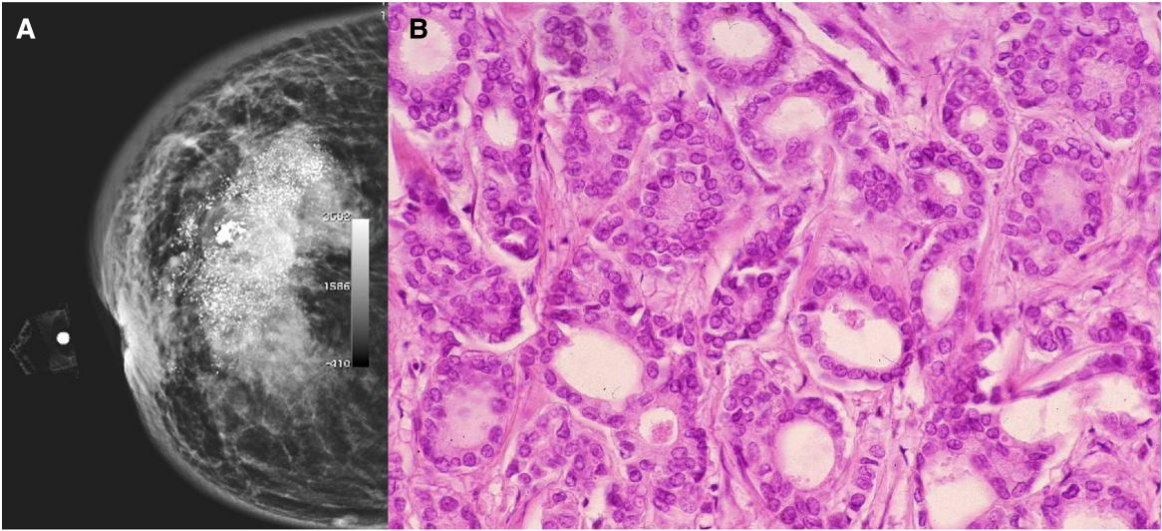
(*A*) Mammography showing a dense mass, heterogeneous, with calcification, with poorly delimited and speculated edges. (*B*) Microscopic image with epithelial neoplastic lesion growing in the ducts with moderate pleomorphism, consistent with invasive ductal carcinoma with a specific pattern.

Mammography showed a dense, heterogeneous mass with ill-defined borders, classified as BIRADS 5 (*Figure 2A*). Subsequently, a biopsy confirmed an infiltrating ductal carcinoma (HER2^+++^, Estrogen^+^ 20%, Progesterone^+^ 90%, Ki67^+^ 30%). The clinical stage was categorized as IIb T4B NX M0 (*Figure 2B*). Metastasis was ruled out, and a prognosis assessment indicated a survival of more than 1 year. However, due to the reduced ejection fraction and high-risk of cardiotoxicity, it was not possible to start oncological treatment. In an interdisciplinary meeting with the medical, surgical, oncological, and interventional cardiology services, it was determined that the patient's life expectancy was > 1 year, that cancer treatment could not be initiated before AS treatment, and that cancer treatment could be delayed for at least 2 months. As a result, it was decided to perform TAVR and medical treatment (for HF with reduced ejection fraction) as a bridging therapy to improve the ejection fraction and, subsequently, begin antineoplastic treatment.

**Figure 3:**
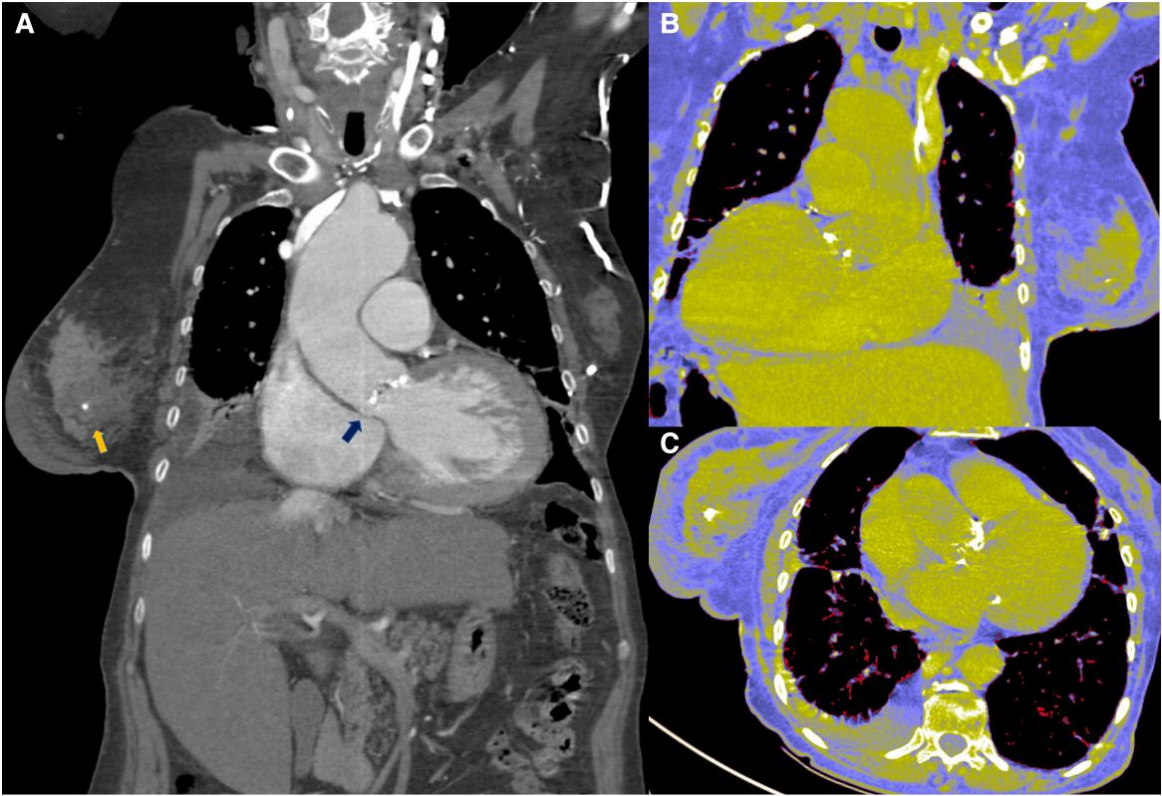
(*A–C*) Tomographic image where both pathologies are observed. Breast tumour and aortic stenosis (arrows).

**Figure 4:**
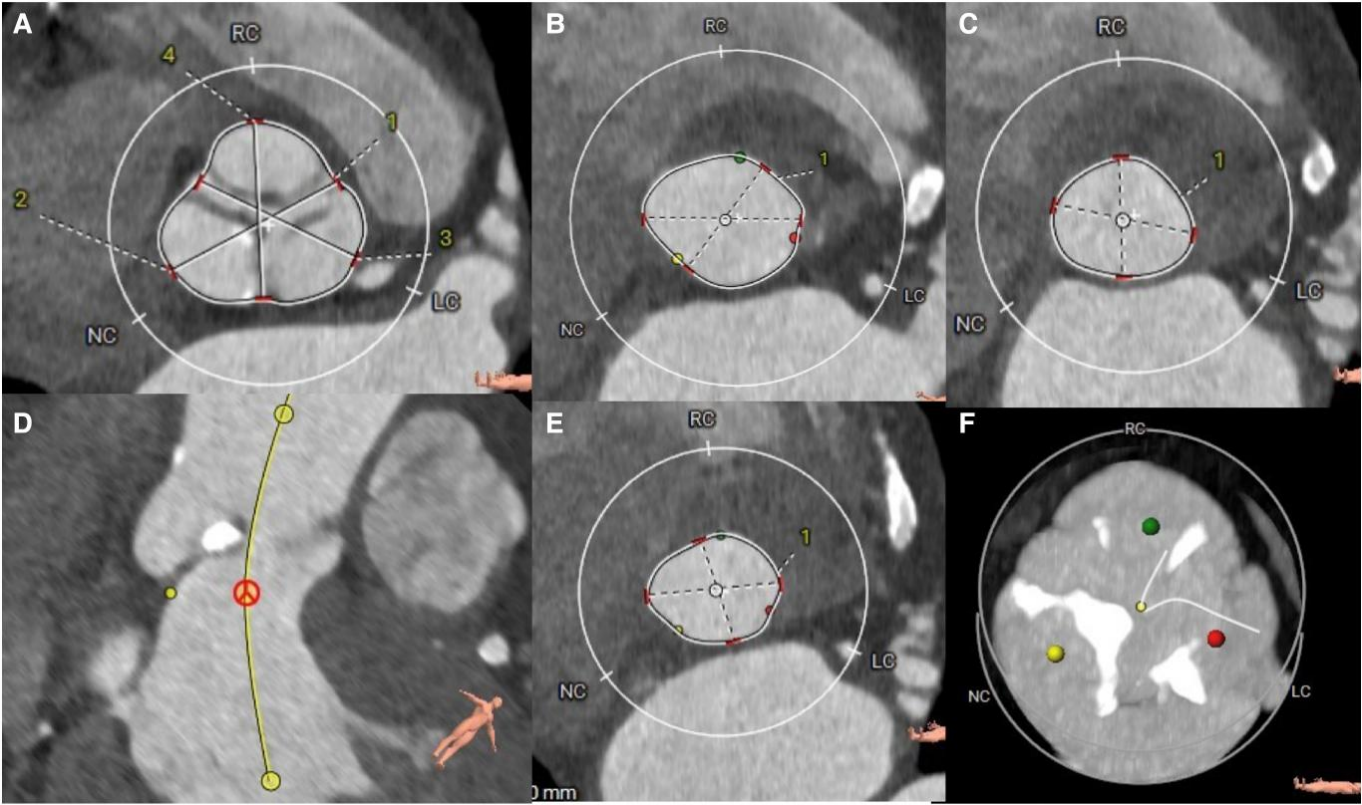
Tomographic planning. (*A*) sinuses of Valsalva, (*B*) aortic annulus in systole, (*C*) annulus in diastole, (*D*) no-coronary sinus, (*E*) outflow tract and, (*F*) degree of moderate calcification. LC sinus, left coronary; RC sinus, right coronary; NC sinus, Non coronary. 1, Right-left commissure; 2, non coronary sinus; 3, left coronary sinus; 4, right coronary sinus.

Concomitant pathology was observed in the thoracic tomography (*Figure 3*). The tomographic planning revealed an aortic valve with tricuspid morphology, moderate calcification, and a left ventricular outflow tract free of calcium (*Figure 4*). The measurements of the aortic annulus were 75 mm in perimeter (*Figure 4*). Based on these results, we decided to place a self-expanding valve, Evolut #29.

**Figure 5:**
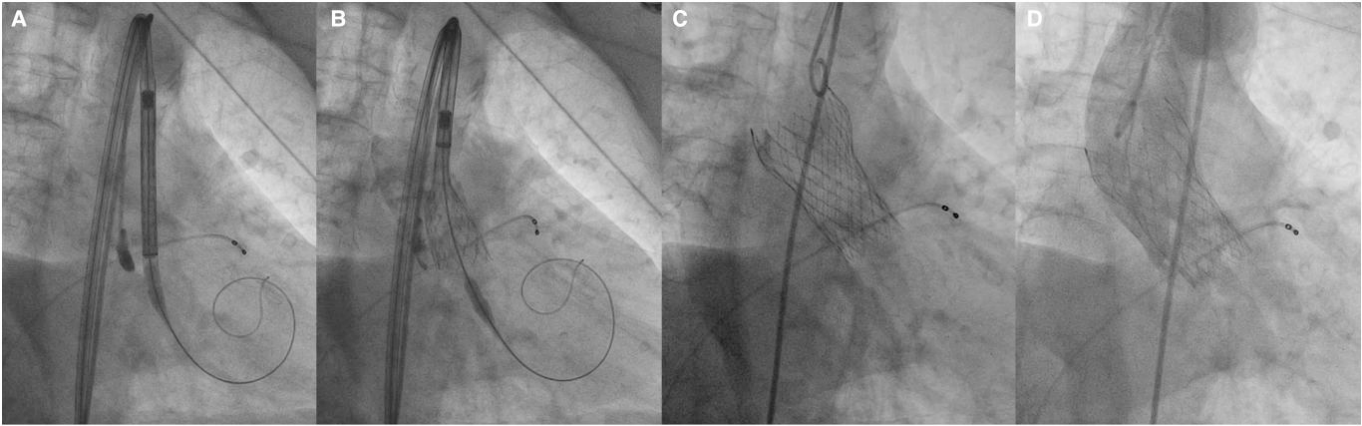
Interventional procedure. (*A* and *B*) Valve alignment, (*C*) complete release of the valve, and (*D*) control aortography, with adequate position and absence of paravalvular leak.

Using a femoral approach, supported by rapid pacing and under sedation, we performed predilation with a 24 × 40 mm balloon. Subsequently, supported by cusp overlap projection and coplanar view, we released the valve without complications. The control aortography demonstrated adequate placement and absence of paravalvular leak (*Figure 5*). Post-interventional transthoracic echocardiography showed a mean gradient of 7 mmHg and a velocity of 1.7 m/s. There were no conduction disorders. She was discharged 48 h after the procedure.

**Figure 6:**
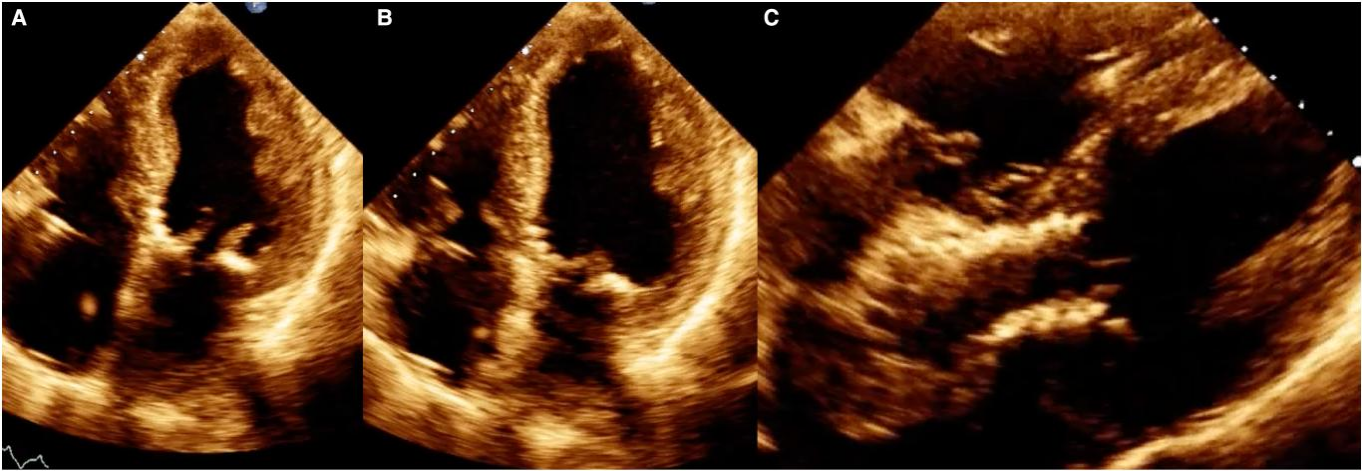
Control echocardiogram 3 months after the procedure. (*A*) Ventricular systole, (*B*) ventricular diastole, and (*C*) image of the percutaneous valve in a modified 5-chamber projection.

At 3 months of follow-up, the echocardiogram showed a significant improvement in the ejection fraction (LVEF 52% PSAP 34 mmHg) and a prosthetic valve without signs of paravalvular leak (*Figure 6* and Supplementary material online, Video S2 of supplementary material). Based on these parameters, the medical oncology service decided to start oncological therapy. The first intervention involved bilateral mastectomy and axillary lymph node resection. The histological report ruled out lymph node infiltration, so curative chemotherapy was started 3 months after TAVR. The chemotherapy included trastuzumab at a dose of 3.6 mg/kg and hormonal therapy with Anastrozole, 1 mg taken orally once a day. Prior to chemotherapy, NT-proBNP and troponin values were 104 pg/mL and 0.01 ng/mL. Due to the high-risk of cardiotoxicity, we decided to continue medical treatment as secondary prevention with Metoprolol 50 mg every 12 h and Enalapril 5 mg every 24 h. The planned chemotherapy treatment was successfully completed with adequate tolerance of myocardial function. Follow-up was conducted by the clinical oncology service, and disease regression was ruled out through PET-CT. Twelve months after the oncology treatment, the disease was classified as being in remission. In the cardiovascular follow-up, he remains in functional class 1, the results of the biomarkers were normal, ventricular function was preserved, although there was a 5% decrease in ejection fraction, it did not meet the criteria for cardiotoxicity.

## Discussion

The simultaneous diagnosis of cancer and severe AS in the same patient is variable. The prevalence varies between 5.4% and 37.8%.^2,6,7,8^ Stachon *et al*.^9^ identified 70 (19%) patients with malignant findings out of 374 patients preoperatively screened for severe AS using computed tomography.

The coexistence of both pathologies is of great importance due to the associated mortality, whether secondary to cancer or due to valvular pathology. Mortality due to HF is more frequently encountered in patients with AS than in cancer patients without aortic valve disease. In a retrospective study, cancer patients with severe AS had a 5-year mortality rate of 48%. 59% of deaths were due to cancer progression, and 31% were due to HF and stroke.^10^

The type of neoplasia associated with AS is variable depending on the individual. In male patients, prostate cancer (42.4%) is the most common, followed by haematological cancer (23.7%) and colon cancer (6.8%). In female patients, breast cancer (35.0%) is the most common, followed by haematological cancer (30.0%) and colon cancer (17.5%).^8^

History of active cancer is conventionally considered a comorbidity limiting patient prognosis. At the same time, in the coexistence of both pathologies, AS may interfere with optimal antineoplastic management (i.e. high-risk oncological surgery or potentially cardiotoxic chemotherapies).^4^ Currently, progress in oncology has turned several malignant tumour types into partially or fully remitted disease. However, whether active cancer affects the prognosis of patients with AS undergoing TAVR remains controversial.^10^

The optimal strategy for managing severe AS in patients with active cancer is utterly difficult to decide. The patients require careful assessment to select the appropriate therapeutic choices and their timing (i.e. valve treatment first vs. cancer treatment first).^4^ A fundamental element to consider in choosing the most suitable type of intervention for the patient, in addition to the prognosis, is the stage of neoplastic pathology. Patients with advanced disease stage, metastases, multiple comorbidities, and very short estimated survival may be candidates for balloon valvuloplasty as a ‘bridge to destination’.^5,10,11^ The expert consensus issued by the Society for Cardiovascular Angiography and Interventions recommends aortic balloon valvuloplasty or TAVR for cancer patients with AS as either palliative therapy or a cure for valvular disease, to improve quality of life or to facilitate appropriate cancer therapy.^11^ Current guidelines suggest intervention only in patients with a life expectancy of at least 1 year.^5^

The decision to ultimately opt for TAVR is not an easy choice and involves a multidisciplinary approach to assessing the appropriateness of intervention.^12^ In our case, due to significant ventricular dysfunction, chemotherapy was contraindicated. Accordingly, we decided to perform TAVR initially to improve the ejection fraction. Subsequently, proceed with the surgical procedure and antineoplastic therapy.

An early improvement in LVEF after TAVR occurs in ∼50% of cases. The reason for left ventricle dysfunction is the afterload mismatch rather than irreversible myocardial damage (due to fibrosis or coexisting coronary artery disease). Consequently, functional improvement is anticipated almost immediately following the procedure.^13^ In patients who undergo TAVR, improvement in ventricular function and regression of myocardial damage has been observed months after valve replacement.^14^ For that reason, transthoracic echocardiography months after the procedure is necessary to assess valve function and structural regression.^5^

Performing TAVR before cancer treatment allows for radical oncologic treatment shortly after valve intervention.^15^ The guidelines on cardiovascular assessment and management of patients undergoing non-cardiac surgery suggest that non-cardiac surgery may be performed early after successful TAVR.^16^ Concerning antineoplastic therapy, the recovery of ventricular function after TAVR should be assessed. In patients with recovery of ventricular function after the procedure, the start of chemotherapy is indicated. By reason of the increasing access of cancer patients to TAVR, delays in cancer treatment have been significantly reduced from about 2 months after cardiac surgery to 2 weeks.^12^ However, the relationship between chemotherapy treatment and the risks associated with cardiotoxicity and HF must be taken into account.^17^

Pre-existing severe valvular heart disease is associated with an increased risk of cancer therapy-related cardiac dysfunction.^17,18^ Among all chemotherapeutic agents, anthracyclines accelerate valve fibrosis, mainly of the left heart.^17^ It is recognized that anti-HER2 therapies may lead to left ventricular dysfunction in up to 15%–20% of patients and to overt HF if surveillance is missed or in high- and very high-risk patients.^18,19^ ACE-I or ARB and beta-blockers recommended for HF should be considered for primary prevention in high- and very high-risk patients receiving anthracyclines and/or anti-HER2 therapies.^20^

In our case, the initiation of treatment with trastuzumab and the association of a high-risk of ventricular dysfunction, the initiation of preventive treatment, and strict monitoring with transthoracic echocardiogram were essential. Fortunately, up to 12 months after the last cycle of chemotherapy, our patient has maintained ventricular function within normal parameters and achieved remission of the neoplastic disease.

In conclusion, due to the exclusion of this type of cases in the guidelines and the absence of sufficient randomized studies, standard treatment is not established. However, for patients with severe AS and concomitant active cancer disease, the TAVR procedure is safe and effective as a bridging treatment prior to antineoplastic management. Individual decision-making is necessary with the support of a multidisciplinary team.

## Supplementary Material

ytae475_Supplementary_Data

## Data Availability

The data underlying this article are available in the article and in its online Supplementary material.

## Lead author biography

Dr Heberto Aquino-Bruno is a clinical cardiologist who graduated from the Centro Medico Nacional 20 de noviembre ISSSTE. He is currently finishing the Fellowship in Interventional Cardiology at the Centro Medico Nacional 20 de noviembre ISSSTE. His main area of interest is coronary and structural intervention. He has made several publications on TAVR.
